# Benefits of a Juvenile Arthritis Support Program (JASP-1) for children recently diagnosed with Juvenile Idiopathic Arthritis and their parents

**DOI:** 10.1186/s41927-024-00404-8

**Published:** 2024-08-15

**Authors:** Karina Mördrup, Johanna Granhagen Jungner, Eva Broström, Karin Palmblad, Cecilia Bartholdson

**Affiliations:** 1 Department of Women´s and Children´s Health, Karolinska Institutet Neuropediatric Unit, Karolinska vägen 37 A, Solna, 171 64 Sweden; 2 Highly Specialized Pediatric Medicine and Orthopedics, Astrid Lindgren Children´s Hospital, Karolinska University Hospital, Stockholm, Sweden

**Keywords:** JIA, Support program, Patient satisfaction, Patient outcome assessment

## Abstract

**Background:**

Medical treatment for children with Juvenile Idiopathic Arthritis (JIA) has improved radically since the development of biological disease-modifying antirheumatic drugs. However, children suffer from pain and anxiety, and parents often experience loneliness and lack of support. Some parents reported that information provided at the time their child was diagnosed could be difficult to assimilate. Therefore, the aim of this study was to develop a Juvenile Arthritis Support Program (JASP-1) for children recently diagnosed with JIA and their parents. Moreover, the aim was to explore patients´ and parents´ experiences with JASP-1 and its potential impact on patients´ physical health.

**Methods:**

JASP-1 included seven patient- and family-centered clinical visit from time of diagnose and one year ahead. Data were collected from a study-specific questionnaire answered by children and their parents after participation in JASP-1 and from the pediatric rheumatology register. The study-specific questionnaire explored participants´ experience with the care they received during their first year with JIA. Registry and questionnaire data from the intervention (JASP-1) group was compared to a control group.

**Results:**

The analysis revealed that children and parents who completed JASP-1 were more satisfied with the care they had received during their first year with JIA than the control group. The results also showed that children who completed JASP-1 were assessed as having better overall health after 12 months, than children in the control group (JASP-1 = mean 4.33, 95% Confidence Interval (CI) 4.17 − 4.46), (Control = mean 3.68, 95% CI 3.29 − 4.06), (*p* = 0.002). Moreover, children in the JASP-1 group had less disease impact on daily life (JASP-1 = mean 0.15, 95% CI 0.07 − 0.24) (Control = mean 0.40, 95% CI 0.13 − 0.67), (*p* = 0.017) and less active joints than the control group (JASP-1 = mean 0.62, 95% CI 0.35 − 1.58), (Control = mean 0.87, 95% CI 0.18 − 1.56), (*p* = 0.054).

**Conclusion:**

A support program like JASP-1 could be an effective way of not only supporting children newly diagnosed with JIA and their parents psychologically but may also increase children’s overall physical health and improve quality of care within pediatric rheumatology.

**Trial registration:**

Retrospectively registered in ClinicalTrials.gov, the 13th of February with ID NCT06284616.

**Supplementary Information:**

The online version contains supplementary material available at 10.1186/s41927-024-00404-8.

## Introduction

Juvenile Idiopathic Arthritis (JIA) is one of the most common diseases acquired during childhood, with symptoms persisting more than 6 weeks and with onset before the age of 16 and with unknown cause [1]. Diagnosis consists of seven categories, all seven of which share inflammatory arthritis as a common denominator. The seven categories; systemic arthritis, polyarthritis rheumatoid factor (RF)-positive, polyarthritis RF-negative, oligoarthritis, enthesitis-related arthritis, psoriasis arthritis, and undifferentiated arthritis are defined by the International League of Association for Rheumatology (ILAR) [1]. In the Nordics, the incidence of children with JIA is approximately 15:100 000 with a prevalence of 1:1–2000 [2].

Medical treatment for children with JIA has improved radically since the development of biological disease-modifying antirheumatic drugs, combined with already existing therapies [3]. However, despite treatment and absence of disease activity, children with JIA still describe problems with pain that could affect quality of life, school attendance, and participation in physical activities [4–6]. A study by Fair et al. (2019) describes that anxiety and depression are more common in children with JIA and their parents compared to healthy peers [7]. Moreover, previous studies shows that parents of children newly diagnosed with JIA often experience loneliness and a lack of information, with few resources available to help them [8]. Some parents report that information provided at the time of diagnosis could be difficult to assimilate [8]. For some, receiving a diagnosis could be a relief, as they now know what the child is suffering from, the prognosis, and the treatment that can be provided [9].

Care for children recently diagnosed with JIA and their parents can differ depending on which hospital they are enrolled in. At the Pediatric Rheumatology Clinic (PRC) at one of Sweden’s largest University Hospitals, standard care entails that children and their parents meet the medical doctor (MD) at their first visit to the PRC. No other profession is in attendance when the child and their parents receive information about the diagnosis and possible treatments. Treatment is often initiated at the first visit. Most families are then scheduled to visit the clinic again for a MD visit approximately 4–6 months after their initial appointment. Sometimes a visit to the physiotherapist is planned during this period. If questions arise during these months, families are referred to the general department, which has limited accessibility.

Few studies are published regarding how best to support children recently diagnosed with JIA and their parents. However, those that have been published suggest that interventions such as education are needed [10, 11]. In a study of children diagnosed with JIA within the last two years, the children and their parent’s received education in the form of either a brochure or a video. Participants gained increased knowledge about disease etiology, treatment, self-care knowledge, and disease relapse management [10].The authors concluded that this indicated a need for the development of educational programs for managing chronic diseases like JIA [10]. A study by André, Hagelberg [11] described an eight-hour educational program attended by adolescents with JIA and their parents. The results showed that participants, especially the parents, improved their competencies for example regarding medication and pain and the authors concluded that an educational program should be a self-evident part of treatment [11]. In addition to educational interventions, psychosocial support is important. A study by Gilljam et al. (2016) showed that children’s participation was promoted by a sense of security and calmness gained through a trusting relationship with healthcare professionals. Children also described the importance of continuity of care, as well as a warm and personal dialogue with healthcare professionals [12]. It is essential that support provided to children and parents is based on their personal needs and on a patient-and family-centered approach. Patient- and family-centered care is described by the Institute for Patient- and Family-Centered Care as “an approach to the planning, delivery, and evaluation of healthcare that is grounded in mutually beneficial partnerships among healthcare professionals, patients, and families” [13]. The use of patient- and family-centered care can help allocate recourses and improve health outcomes [14].

Previously mentioned evidence suggests that interventions to improve knowledge and participation could facilitate families in coping with the novel and stressful situation when a child receives a JIA diagnosis. Furthermore, clinical experience from caring for newly diagnosed children and their parents provides empirical evidence demonstrating that families often leave the PRC with feelings of stress and anxiety.

## Methods

### Aim

The aim of this study was to develop a Juvenile Arthritis Support Program (JASP-1) for children recently diagnosed with JIA and their parents. Moreover, the aim was to explore patients´ and parents´ experiences with JASP-1 and its potential impact on patients´ physical health.

### Research questions

- What are the differences in experiences from children and parents participating in JASP-1 compared to a control group?
- Are there differences in perceptions of how much JIA impacts children’s daily life between children participating in JASP-1 and the control group, and if so, to what extent?
- Is there a difference in joint activity between children participating in JASP-1 and those in the control group, and if so, to what extent?

### Study design

**Fig. 1:**
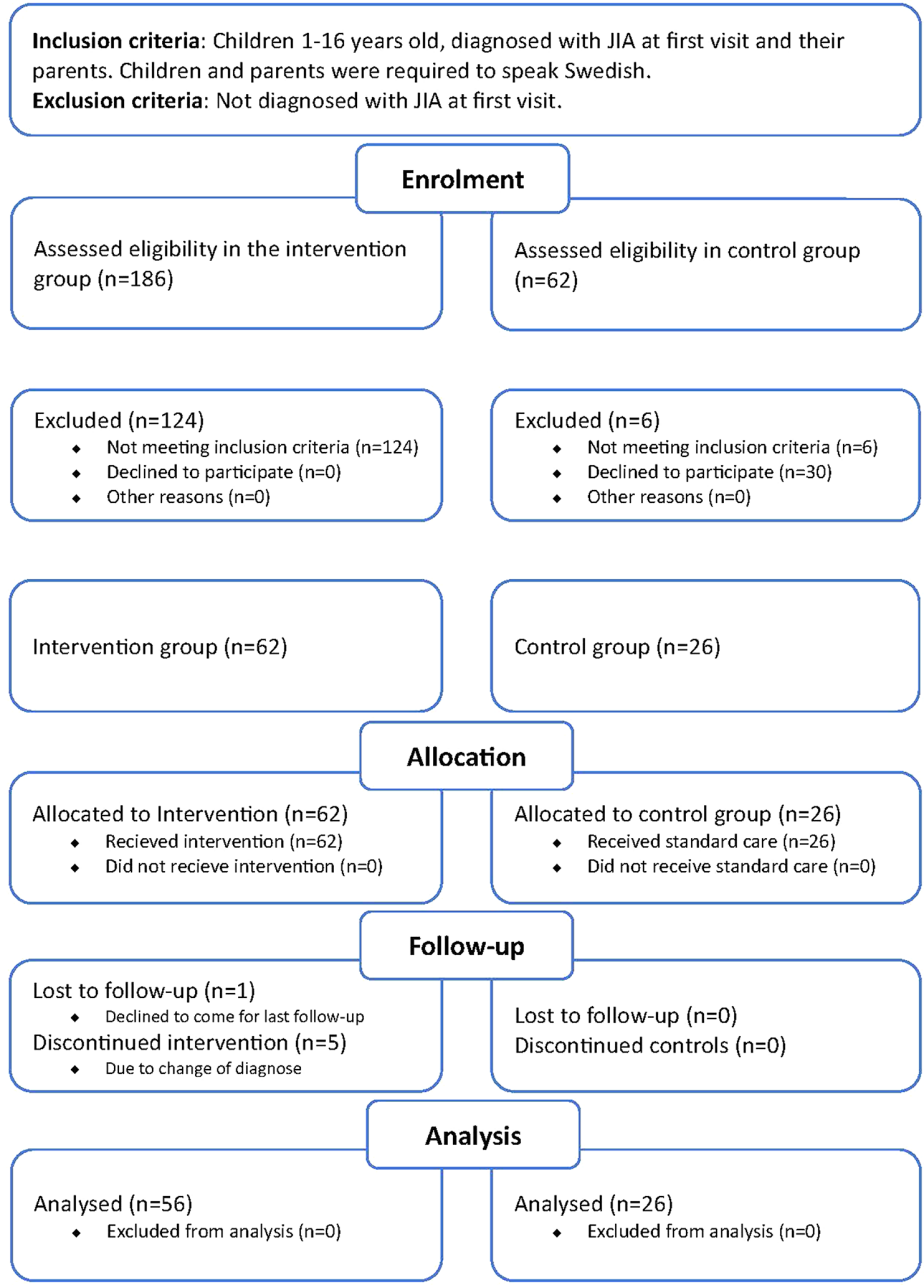
CONSORT flow chart

The design of the study was a (non-randomized quasi-) intervention study (Fig. 1).

#### Development of JASP-1

The development of JASP-1 was conducted in collaboration with the interprofessional rheumatology team at the University Hospital, including six medical doctors (MD), four registered nurses (RN), three of these holding pediatric specialist training, one nursing assistant (NA), two physiotherapists (PT), one occupational therapist (OT), and one external organizational coach. The organizational coach was funded by a pharmaceutical company with the aim of providing support in improving care. In addition to the interprofessional rheumatology team two research partners, representing patients, were involved in the project. The research partners had own experience with JIA and were diagnosed during childhood. Their role was to discuss and give feedback on the structure of the JASP-1.

Five workshops were held over the course of one year. The first workshop was held in May 2017 and the last was held in March 2018. Each meeting lasted 2–3 h. At the end of every meeting, the team, together with the coach, planned individual work until the next meeting and assigned it; for example, instructions to outline a draft of the template for RN visits. At the following meeting, individual work was presented, and after consensus merged into the program. The JASP-1 was developed in stages, from the arrival of referrals to end of the children’s first year with a JIA diagnosis. More details of the development of JASP-1are illustrated in supplementary materials.

A JASP-1 pilot was conducted (2018–2019) with eight children recently diagnosed with JIA and their parents, to investigate the program’s feasibility and content. Content validity was ensured by receiving input from the children and the parents to ensure that the content was meaningful and relevant to them. Revision of the program was not considered needed after the pilot, and the structure of the JASP-1 was determined according to the study protocol.

#### Structure of JASP-1

To be able to conduct this study, adjustments were needed regarding how the referrals were assessed by the MD specialized in pediatric rheumatology. If the MD assessed the child as suffering from JIA, the referrals were marked “+ RN”. This resulted in an RN, who was well acquainted with the program, attending when the child and parents came for their first visit.

Those diagnosed with JIA were invited to participate in the JASP-1. The structure of the visits (Table 1) and coordination of the interprofessional rheumatology team were led by the RN.

**Table 1:** The JASP-1 program included seven patient- and family-centered clinical visits

Visit	Week	Duration	Participants	Content
1	Visit week 0	One hour with MD and RN and 45 min with the RN	Child Parent(s) MD RN	The MD and RN met the child and the parent(s) together. According to the patient- and family-centered care approach, the RN communicated with the child and parent(s) after the joint visit. The aim was to answer questions, get to know the family, and ensure they have understood the information and diagnosis. The family received a direct telephone number to the RN.
2	Team visit week 2–4	One hour with each profession, i.e., 3 h	Child Parent(s) RN PT OT	Introduction to the team. The RN communicated with the families about the diagnosis, answered their questions, and made sure they understood the information they had been given and the treatment plan. The PT made a careful joint assessment and talked about physical activity, and the OT assessed hand functionality and daily life activities. Support was offered according to child and parent needs; for example, regarding their feelings after diagnosis, and how and when to contact the PRC.
3	Visit or phone week 3–8	30 min	Child Parent(s) RN	According to child and parent preferences, the visit was held at the clinic or by phone. The RN explored how the family was doing and answered their questions. Support was offered according to the child’s and parents´ needs; for example, regarding worries about the future after a JIA diagnosis.
4	Visit week 12	45 min	Child Parent(s) MD RN	The MD and RN assessed together how the child was doing, the potential effect of medication, and explored how the family was coping. Support was offered according to the child’s and parents’ needs; for example, regarding how to handle subcutaneous injections at home.
5	Visit or phone week 14–18	30 min	Child Parent(s) RN	According to child and parent preferences, the visit was held either at the clinic or by phone. The RN explored how the family was doing and how medication administration worked as well as effects and eventual side-effects. The RN also answered the children’s and parents’ questions. Support was offered according to child and parent needs; for example, about side effects of medication.
6	Visit week 26	45 min	Child Parent(s) MD RN	The MD and RN assessed together how the child was doing, the potential effect of medication, and explored how the family was coping. Support was offered according to child and parent needs; for example, conversation about coping strategies.
7	Visit week 52	45 min	Child Parent(s) MD RN	The MD and RN assessed together how the child was doing, the potential effect of medication, and explored how the family was coping. Support was offered according to child and parent needs; for example, a conversation about the child’s medication.

#### Participants

##### Inclusion criteria

Children diagnosed with JIA, including all categories, at their first visit to the PRC and their parents. The criteria for a JIA diagnosis are: the child has to be younger than 16 years old and have inflammation in one or more joints which persists for more than 6 weeks [1]. Other reasons for arthritis were ruled out by blood test before arrival at the PRC. Both children and their parents were required to speak Swedish. Due to the COVID-19 pandemic participants were recruited from 15th of August 2019 to 15th April 2022. Sample size was directed by the number of children diagnosed with JIA at the PRC during one year, i.e. approximately 50 children per year.

##### Exclusion criteria

Children that did not fulfil the criteria for JIA at the first visit.

##### Control group

The control group were identified from a list at the PRC containing children diagnosed with JIA after the first visit. Children and parents in the control group were invited to participate at the time for their 12 months follow-up with the MD. If not visiting the PRC, study participation (questionnaire) was offered by post. The control group consisted of children 1–16 years old, diagnosed with JIA at the first visit and their parents. The control group received standard care and most of these children were diagnosed before the start of JASP-1 and were therefore not included in the intervention (Fig. 1).

### Measures

#### Study specific satisfaction questionnaire

To measure the children’s and parents’ experiences with the care they had received during the first year with JIA, the children and parents who were included in JASP-1 were invited to answer a study-specific questionnaire in connection to their 12-month visit to the PRC. To enable identification of differences in experiences with care, the control group was invited to answer the same study-specific questionnaire. The study-specific questionnaire measuring patient experience was composed in collaboration with the Swedish Association of Local Authorities and Regions. The study-specific questionnaire included 16 questions in Swedish aiming to measure children’s and parent’s experiences of the care they had received during their first year with JIA regarding the following domains: information, communication, participation, and emotional support. One example of a question was: “If you/ your child asked questions to healthcare professionals, did you/your child get answers that you/your child understood?” Response alternatives ranged from “No, not at all” to “Yes, completely” on a 5-point Likert scale. At the end of the questionnaire, one question was formulated as, “How do you assess your/your child’s overall health condition?” with response options ranging from “Not good at all” to “totally good.” Children younger than 8 were assisted by their parents in answering the study-specific questionnaire, and children between 8 and 17 could choose to answer independently or together with their parents.

#### The Swedish pediatric rheumatology quality register

The Swedish Pediatric Rheumatology Quality Register (PedSRQ) [15] is a national quality registry which started in 2009 and includes children with JIA throughout Sweden. In the registry, information about treatment, disease- and joint activity (registered by MD or RN), and patient-reported outcome measures (PROM), are measured. The PROM was registered by the child or the parents, depending on the child’s age, and measured with the disease-specific Child Health Activity Questionnaire (CHAQ), which assesses functional ability and assists in understanding the impact of the disease on the child´s daily life. The total CHAQ-score, as well as active joints and treatment at 12 months was registered in the PedSRQ. Demographics of children in both the JASP-1 group and the control group was collected from PedSRQ. For the control group, CHAQ and number of active joints were collected retrospectively 12 months after diagnose.

### Data analysis

Data analysis was performed using IBM SPSS Statistics for Windows, version 28 (IBM Corp., Armonk, N.Y., USA). Demographics of the children as well as children´s and parents’ responses to the questions were analyzed using descriptive statistical analysis (frequency, percentage, and median. Comparative analyses were made using non-parametric tests (Mann Whitney U test) with a significance level (p-value) set to < 0.05.

## Results

During the study period (August 2019–April 2022) the RN and MD assessed 186 children, who, according to their referral, had a potential JIA diagnosis, of which 62 were diagnosed with JIA.

### Demographics of children included in JASP-1 and the control group

All children and parents who were invited to participate in JASP-1 accepted the invitation. Initially 62 children were included in JASP-1, six were excluded, resulting in 56 children and their parents completing JASP-1. The exclusions were due to five changes in diagnosis during the first year and one participant who dropped out before the final visit. The demographics of children included in JASP-1 and in the control group are shown in Table 2.

**Table 2:** Description of participants’ age, gender, JIA categories, and treatment. Treatment is described at 12 months

Demographics	JASP-1, *n* = 56	Control group, *n* = 26
Age range at diagnose (median)	1–16 (10)	1–16 (9.5)
**Gender**	**% (n)**	**% (n)**
Female	64.3 (36)	76.9 (20)
Male	35.7 (20)	23.1 (6)
**JIA categories**		
Polyarthritis RF pos	10.7 (6)	3.8 (1)
Polyarthritis RF neg	19.6 (11)	19.2 (5)
Oligoarthritis	35.7 (20)	30.8 (8)
Psoriatic arthritis	3.6 (2)	3.8 (1)
Enthesitis-related arthritis	12.5 (7)	0
Undifferentiated arthritis	10 (17.9)	34.6 (9)
**Treatment at 12 months**		
No treatment*	21.4 (12)	19.2 (5)
Methotrexate (MTX)	25 (14)	30.8 (8)
Biologic	8.9 (5)	11.5 (3)
MTX and biologic	44.6 (25)	38.5 (10)

* The “No treatment” was agreed upon between the MD and the patient/parent’s and indicates that no active disease was observed

### Demographics of responders to the study-specific questionnaire

In both groups the participants could, if the child was above eight years old, choose whether the child and the parents wanted to answer the questionnaire together or separately. For children under eight, the parents answered alone or together with the child. For the JASP-1 group, the response frequency to the questionnaire was 100%, and for the control group, the response frequency was 46%. Of the answers in the JASP-1, 51% (40) were from parents, 43% (34) were from children, and 6% (5) chose to answer together. In the control group, 61% (20) of the questionnaires were answered by parents, 27% (9) by children, and 12% (4) answered together. Parents’ education level was the same in both groups with approximately 12% having graduated from high school and 88% having graduated from university. In the JASP-1, 89% of parents lived together compared with 83% who lived together in the control group.

### Children’s and parents’ responses to the study-specific questionnaire

Analyses revealed that both children and parents, as well as shared responses in JASP-1 rated higher scores in all answers except one, which was “Did the HCP treat you/your child with compassion and care?” which was equal with the control group. In 10 of the 16 answers, there were a significant difference (marked as * in Table 3). Higher scores meant higher levels of satisfaction. Some of the significant answers pertained to whether they felt they had received enough information about how the health condition could affect the everyday life, where to turn to for help, or where to ask questions after the diagnosis, and whether they have had the opportunity to get emotional support when needed. The participants in JASP-1 assessed the children’s overall health condition as better than the control group.

**Table 3:** Children and parent’s responses to the study specific questions about satisfaction with care, both JASP-1 and control group presented with mean values, CI and P-values

Questions	JASP-1 (95% CI)	Control (95% CI)	*P*-values
Were the healthcare professionals (HCP) well versed in your/your child’s health record? *	4.82 (4.73 − 4.91)	4.48 (4.3 − 4.74)	0.010*
Did the HCP take your/your child’s experiences of the health condition into account? *	4.91 (4.83 − 4,.9)	4.70 (4.53 − 4.74)	0.001*
Did the HCP treat you/your child with compassion and care?	4.94 (4.88 − 4.99)	4.94 (4.85 − 5.03)	0.988
If you/your child spoke with several HCP, were they consistent in their communication? *	4.77 (4.64 − 4.90)	4.50 (4,21 − 4.79)	0.029*
Did you/your child get the opportunity to ask the questions you wished?	4.95 (4.90 − 5.0)	4.85 (4,.72 − 4.98)	0.078
If you/your child asked questions of the HCP did you/your child, get answers you understood? *	4.86 (4.76 − 4.96)	4.58 (4.36 − 4.79)	0.002*
If the HCP spoke with each other about you/your child, were you/your child included in the conversation?	4.88 (4.80 − 4.96)	4.63 (4.32 − 4.94)	0.108
Were you involved in the decisions regarding your/your child’s care and treatment to the extend you wished?	4.79 (4.70 − 4.89)	4.75 (4.57 − 4.93)	0.726
Did you/your child receive sufficient information about how the health condition can affect the everyday life? *	4.74 (4.62 − 4.87)	4.28 (3.97 − 4.59)	0.001*
Did you/your child receive enough information about where to turn to for help if you had additional questions after the visit? *	4.79 (4.68 − 4.91)	4.10 (3.67 − 5.53)	< 0.001*
Did the HCP explain the treatment/medication in a way you/your child understood?	4.80 (4.69 − 4.90)	4.61 (4.30 − 4.92)	0.463
If you/your child felt uncomfortable about the health condition or medication, were you met with compassion and care?	4.83 (4.72 − 4.94)	4.69 (4.42 − 4.96)	0.290
Did you/your child have the opportunity to get emotional support from the HCP if necessary? *	4.75 (4.57 − 4.92)	4.14 (3.35 − 4.56)	< 0.001*
Would you recommend the PRC to others in the same situation? *	5.0 (5.9 − 5.0)	4.91 (4.80 − 5.01)	0.006*
Do you believe that the PRC coordinated the visits to the extend you needed? *	4.85 (4.76 − 4.93)	4.27 (3.94 − 4.59)	< 0.001*
How do you assess your/your child’s health condition? *	4.33 (4.17 − 4.49)	3.68 (3.29 − 4.06)	0.002*

* *Significant, p-value < 0.05*

#### Children´s responses to the study specific questionnaires

Within the group of children responding to the questionnaire, 34 had participated in JASP-1 and 9 were from the control group. Children participating in JASP-1 rated higher scores (higher satisfaction) in 13 of the 16 answers, than the children in the control group (presented in Table 4). The children completing JASP-1 experienced that they had received enough information about where to turn to for help and they assessed their overall health condition as better than the children in the control group. Due to the low frequency in the control group, significant differences have not been calculated.

**Table 4:** Children’s responses to the study specific questions about satisfaction with care, both JASP-1 and control group presented with mean values and CI

Questions	JASP-1 (95% CI)	Control (95% CI)
Were the healthcare professionals (HCP) well versed in your health record?	4.82 (4.68 − 4.96)	4.67 (4.12 − 5.21)
Did the HCP take your experiences of the health condition into account?	5.0 (5.0–5.0)	4.89 (4.63 − 5.15)
Did the HCP treat you with compassion and care?	4.94 (4.86 − 5.02)	5.0 (5.0–5.0)
If you spoke with several HCP, were they consistent in their communication?	4.74 (4.47 − 5.0)	4.57 (3.52 − 5.62)
Did you get the opportunity to ask the questions you wished?	5.0 (5.0–5.0)	4.89 (4.63 − 5.15)
If you asked questions of the HCP did you, get answers you understood?	4.82 (4.61 − 5.03)	4.44 (3.89 − 5.0)
If the HCP spoke with each other about you, were you included in the conversation?	4.84 (4.70 − 4.98)	4.17 (2.77 − 5.56)
Were you involved in the decisions regarding your care and treatment to the extend you wished?	4.79 (4.64 − 4.94)	5.0 (5.0–5.0)
Did you receive sufficient information about how the health condition can affect the everyday life?	4.79 (4.56 − 5.02)	4.25 (3.51 − 4.99)
Did you receive enough information about where to turn to for help if you had additional questions after the visit?	4.79 (4.63 − 4.96)	3.57 (2.17 − 4.97)
Did the HCP explain the treatment/medication in a way you understood?	4.88 (4.77 − 5.0)	4.88 (4.58 − 5.17)
If you felt uncomfortable about the health condition or medication, were you met with compassion and care?	4.84 (4.67 − 5.01)	5.0 (5.0–5.0)
Did you have the opportunity to get emotional support from the HCP if necessary?	4.77 (4.56 − 4.98)	4.50 (3.62 − 5.38)
Would you recommend the PRC to others in the same situation?	5.0 (5.0–5.0)	4.88 (4.58 − 5.17)
Do you believe that the PRC coordinated the visits to the extend you needed?	4.94 (4.85 − 5.03)	4.38 (3.61 − 5.14)
How do you assess your health condition?	4.26 (3.96 − 4.57)	3.13 (2.30 − 3.95)

#### Parents´ responses to the study specific questionnaires

A total of 60 parents responded to the study-specific questionnaire, 40 participating in JASP-1 and 20 from the control group. In all answers except one, where they were equal the parents from JASP-1 rated higher scores than the parents from the control group. In six of the answers, there were significant differences between the groups (marked as * in Table 5). The parents from JASP-1 experienced, for example, that they received more information and emotional support than the ones in the control group, and they also assessed their child’s overall health condition to be better.

**Table 5:** Parents’ responses to the study specific questions about satisfaction with care, both JASP-1 and control group presented with mean values, CI and P-values

Questions	JASP-1 (95% CI)	Control (95% CI)	*P*-values
Were the healthcare professionals (HCP) well versed in your child’s health record?	4.82 (4.96 − 4.95)	4.55 (4.23 − 4.87)	0.112
Did the HCP take your experiences of the health condition into account?	4.85 (4.70 − 5.0)	4.75 (4.54 − 4.96)	0.164
Did the HCP treat you and your child with compassion and care?	4.93 (4.84 − 5.01)	4.90 (4.76 − 5.04)	0.743
If you spoke with several of the HCP, were they consistent in their communication?	4.79 (4.66 − 4.93)	4.59 (4.27 − 4.91)	0.209
Did you get the opportunity to ask the questions you wished?	4.90 (4.80 − 5.0)	4.90 (4.76 − 5.04)	1.000
If you asked questions of the HCP, did you get answers you understood?	4.88 (4.77 − 4.98)	4.70 (4.48 − 4.92)	0.101
If HCP spoke with each other about your child, were you included in the conversation?	4.90 (4.77 − 5.02)	4.71 (4.40 − 5.01)	0.106
Were you involved in the decisions regarding your child’s care and treatment to the extend you wished?	4.77 (4.62 − 4.93)	4.75 (4.49 − 5.01)	0.964
Did you/your child receive sufficient information about how the health condition can affect the everyday life? *	4.70 (4.53 − 4.87)	4.25 (3.82 − 4.68)	0.045*
Did you/your child receive enough information about where to turn to for help if you had additional questions after the visit? *	4.77 (4.60 − 4.94)	4.21 (3.69 − 4.73)	0.015*
Did the HCP explain the treatment/medication in a way you and your child understood?	4.70 (4.52 − 4.88)	4.42 (3.93 − 4.91)	0.453
If you/your child felt uncomfortable about the health condition or medication, were you met with compassion and care?	4.84 (4.67 − 5.0)	4.53 (4.12 − 4.93)	0.062
Did you and your child have the opportunity to get emotional support from the HCP if necessary? *	4.72 (4.43 − 5.01)	3.38 (2.44 − 4.33)	< 0.001*
Would you recommend the PRC to others in the same situation? *	5.0 (5.0–5.0)	4.90 (4.76 − 5.04)	0.044*
Do you believe that the HCP at the PRC coordinated the visits to the extend you needed? *	4.77 (4.64 − 4.91)	4.28 (3.83 − 4.72)	0.019*
How do you assess your child’s health condition? *	4.37 (4.19 − 4.56)	3.70 (3.29 − 4.29)	0.031*

* Significant, p-value < 0.05

#### CHAQ and active joints

There were no significant differences discovered in CHAQ and active joints between the JASP-1 group and the control group when the children were diagnosed. However, after 12 months, there was a significant difference in CHAQ and close-to-significant difference in active joints between the groups (Table 6), meaning that children in JASP-1 perceived less disease impact on daily life and had fewer active joints than the control group.

**Table 6:** Mean, 95% CI and p-values for CHAQ and number of active joints in JASP-1 and the control group at diagnose/12 months

	JASP-1	Control group	
**CHAQ**	**Mean (SD) (** ***n*** **)** **(95% CI)**	**Mean (SD) (** ***n*** **)** **(95% CI)**	***P*** **-value**
At diagnosis	0.68 (0.58) (*n* = 49) (0.51 − 0.84)	0.72 (0.82) (*n* = 12) (0.17 − 1.27)	0.781
At 12 months	0.15 (0.29) (*n* = 48) (0.07 − 0.24)	0.40 (0.54) (*n* = 19) (0.13 − 0.67)	0.017**
**Active joints**	**Mean (SD) (** ***n*** **)** **(95% CI)**	**Mean (SD) (** ***n*** **)** **(95% CI)**	**P-value**
At diagnosis	5.64 (6.48) (*n* = 53) (3.85 − 7.43)	6.11 (6.48) (*n* = 19) (2.98 − 9.23)	0.624
At 12 months	0.62 (3.47) (*n* = 50) (0.35 − 1.58)	0.87 (1.6) (*n* = 23) (0.18 − 1.59)	0.054*

^**^ Significant

^*^Close to significant

## Discussion

In this study, the aim was to develop and implement JASP-1 and to explore whether a patient- and family-centered program like JASP-1 could affect children´s and parent’s perceptions of disease impact on daily life and active joints as well as experience with the care, support, and information they received during their first year with JIA. This was done by analyzing data from the pediatric rheumatology quality register and comparing outcomes from the study-specific questionnaire answered by the children and parents completing JASP-1 with children and parents who had received standard care.

The two groups were equal in age, but with a slight difference in some of the JIA categories, with more polyarticular RF + in JASP-1 (10.7%) than in the control group (3.8%). We have observed that the rate of RF-positive polyarthritis in JASP-1 is slightly higher than the normal rate in the Nordic countries. Also noted are the number of children with undifferentiated arthritis, at 10% in JASP-1 and 34.6% in the control group. In both JASP-1 and in the control group there were more girls diagnosed with JIA than boys. However, this is in line with the gender distribution among children diagnosed with JIA in Sweden [16]. The findings reported here, suggest that the groups are comparable.

Analysis of answers from children and parents in JASP-1 compared to answers from children and parents in the control group revealed that the ones completing JASP-1 overall assessed higher levels of satisfaction. This indicate that the children and parents who completed JASP-1 experienced that through the patient- and family-centered care in JASP-1, they had received more information and emotional support than the families in the control group and were therefore more satisfied with the care they had received. These results are consistent with the findings from a telephone intervention that showed increased satisfaction with care after contact with an RN [17]. Even though it may not be very surprising that children and parents were more satisfied with care when they had received patient-centered support for one year after diagnosis, it is interesting to note that the difference was present in almost all items and in all domains.

When analyzing CHAQ and active joints collected from the PedSRQ, the results showed no significant differences at diagnosis between the two groups. However, it was a significant difference in CHAQ and close to a significant difference in active joints between the two groups after 12 months. The children completing JASP-1 had, after 12 months, less active joints assessed by the MD and lower (better) CHAQ values than the children in the control group. These findings are also in line with the results from the study regarding the impact of a nurse-led telephone intervention, which showed a positive impact on pain and morning stiffness when families had the possibility of consulting a RN [17]. We would argue that participating in JASP-1 gives patients, their parents and healthcare professionals more opportunities to detect active joints earlier in the course of the disease and in this way, it is possible to adapt treatment regimens earlier which could explain the differences between the groups. We also believe that the disease’s impact on daily life is affected by the child and family receiving patient-and family-centered support on how to deal with the various challenges that living with JIA entails. The present study raises the possibility that a support program like JASP-1 could play an important role for the child’s well-being; not only psychosocial but also physical. In summary, it seems that JASP-1 can help increase the children’s overall health, but to ensure statistical validity larger studies are needed.

The children completing JASP-1 were perceived to be overall healthier than the children from the control group. This is confirmed both in the questionnaire and by significant differences in the self-assessed CHAQ as well as the MD-assessed fewer active joints at 12 months for the ones completing JASP-1. One could argue that perception of health is dependent on not only physical health, but also on psychological and social health. The concept of health has, in recent decades, been described as a “state of complete physical, mental, and social well-being, not merely the absence of disease or infirmity” [18]. However, it is debated that the definition must be rediscovered, since the population is aging and more individuals are living with chronic illnesses [19]. We contend that the perception of health is dependent on the holistic view of humans and that children with chronic illnesses, such as JIA, could experience good health even though they are not physically well but experience good psychological and social health. The JASP-1 is an example of how to support children and their parents to optimize and create conditions for good psychological and social health, thereby improving their overall perception of health.

Moreover, patient- and family-centered care were used in JASP-1 and made it possible to adapt visits and communication between visits, according to each family’s needs [13]. It is possible that JASP-1’s inclusion of patient- and family-centered care also can explain a part of why the patients completing JASP-1 were perceived overall healthier than the children in the control group after 12 months. It might be that the ones completing JASP-1 understood the disease better (JIA), had better compliance, reached out to the RN with questions, and received support when needed, and therefore felt safer in their situation. Previous research has shown that anxiety and depression are common among children with JIA and their parents [7]. This could be because of lack of understanding of the disease and support. The overall results from this study match well with previous research, which has indicated that the care for children with JIA is in need of improvement. The improvement specially regards the information and support given to the patient and to parents, as well as the communication in the time between visits [20]. One way of improving this is to offer children and their parents a support program like JASP-1 when they are diagnosed with JIA.

### Strengths and study limitations

A strength of this study is that 56 children, representing different ages, and their parents successfully completed JASP-1 during the study period. However, a limitation is that fewer children and their parents participated in the control group. When investigating who answered the study-specific questionnaire it shows the proportion of children 8–15 years old who answered the study-specific questionnaire in the JASP-1 group were larger than in the control group. The increased contact and continues support with the patient’s and parents in JASP-1 and less contact with the patients and parents in the control group could explain why the response rate from the control group was 46% against 100% in the JASP-1.

Randomization of the study was initially discussed to enhance credibility, but according to standards from the Swedish Ethical Review Authority all children/parents must be informed about the randomization and consent to participate before the planned first visit. This information should then have been in the public waiting room, before the MD had assessed whether or not the child had a JIA diagnose. This arrangement was viewed as not ethically defendable and problematic practically. Thus, using historical controls, that were cared for at the same clinic with the same healthcare professionals, was deemed to be the best option.

A limitation of this study is the uncertainty whether or not the different distribution of JIA categories in the two groups could have affected the result. A further limitation of the study is the unknown effect of how the COVID-19 pandemic affected the children and their parents. When working with the study-specific questionnaire, after the JASP-1 participants and the control group answered the questions, we became aware that some of the questions were a little unclear in the formulation. It would have been better to split the questionnaire into a parental and a child part. Additionally, it would have been interesting if there were possibilities to collect data about the child’s age, gender, and diagnosis on the study-specific questionnaire to be able to investigate whether there were any differences in the answers related to the children’s age, gender, or diagnosis. To obtain a deeper understanding of experiences participating in the JASP-1, qualitative interviews need to be performed with children and parents who have completed the program.

## Conclusion

The findings of this study can be understood as JASP-1 had a positive impact on children and parents’ satisfaction with care. Moreover, it seems that children who participated in JASP-1 experienced a positive effect on their disease impact on daily lives and affected joints. Additionally, JASP-1 also seems to have played an important role for children´s and parent´s perceptions of the children´s overall health. Based on these findings, we conclude that the JASP-1 is likely an effective way to support children and parents after JIA diagnosis and has the potential to improve patient-and family centered quality of care in pediatric rheumatology.

### Clinical implications

Implementing a comprehensive support program for children recently diagnosed with JIA is beneficial to address their physical, emotional, and psychosocial well-being. The results from the study can be used to implement JASP-1 nationwide to enable equivalent care in Sweden and most likely in other countries. The results could also be used to strive for cost-effective care because deterioration in the children will probably be detected earlier, and in this way the outcome of the disease can hopefully be improved and thereby decrease the need for hospital visits.

## Electronic supplementary material

Below is the link to the electronic supplementary material.

Supplementary Material 1

## Data Availability

Data is provided within the manuskript or supplementary information files.

## Abbreviations

JASP-1: Juvenile Arthritis Support Program during one year
JIA: Juvenile Idiopathic Arthritis
ILAR: International League of Association for Rheumatology
PRC: Pediatric Rheumatology Clinic
HCP: Healthcare Professionals
MD: Medical Doctor
RN: Registered Nurse
NA: Nursing Assistant
PT: Physio Therapist
OT: Occupational Therapist
PedSRQ: The Swedish Pediatric Rheumatology Quality Register
PROM: Patient Reported Outcome Measures
CHAQ: Child Health Activity Questionnaire
