# Supplementary material for: Benefits of a Juvenile Arthritis Support Program (JASP-1) for children recently diagnosed with Juvenile Idiopathic Arthritis and their parents
Source: BMC Rheumatol. 2024 Aug 15;8:35. doi: 10.1186/s41927-024-00404-8 (PMC11325655; doi:10.1186/s41927-024-00404-8)
Supplement: Supplementary file 1 — Supplementary Material 1 [file 41927_2024_404_MOESM1_ESM.docx]

*Supplementary material: Development of JASP-1*
